# Antioxidant-Induced Stress

**DOI:** 10.3390/ijms13022091

**Published:** 2012-02-16

**Authors:** Cleva Villanueva, Robert D. Kross

**Affiliations:** 1 Escuela Superior de Medicina del IPN, Posgrado e Investigacion, Plan de San Luis y Salvador Diaz Miron S/N, Colonia Casco de Santo Tomas, Mexico, DF. 11340, Mexico; 2 Kross-Link Laboratories, Bellmore, NY 11710, USA; E-Mail: krosslink@aol.com

**Keywords:** antioxidant, oxidative stress, harmful effects

## Abstract

Antioxidants are among the most popular health-protecting products, sold worldwide without prescription. Indeed, there are many reports showing the benefits of antioxidants but only a few questioning the possible harmful effects of these “drugs”. The normal balance between antioxidants and free radicals in the body is offset when either of these forces prevails. The available evidence on the harmful effects of antioxidants is analyzed in this review. In summary, a hypothesis is presented that “antioxidant-induced stress” results when antioxidants overwhelm the body’s free radicals.

## 1. Introduction

The emergence of aerobic organisms was a consequence of oxygen becoming a significant component of the earth’s atmosphere. The evolution of aerobic organisms was probably the result of their gradual adaptation to oxygen. Antioxidant enzymes probably facilitated part of such adaptation, since strict anaerobes have neither superoxide dismutase (SOD) nor catalase, whereas aerotolerant anaerobes have small levels of those enzymes [1]. Aerobic organisms use oxygen to produce the chemical energy they need to survive. Metabolic pathways of aerobic organisms lead to the obligate production of reactive oxygen species (ROS) and reactive nitrogen species (RNS), which are not necessarily bad. Indeed, reactive species are actually needed as signal transduction elements in processes that are essential for life, such as cell growth, differentiation, preconditioning and cell death [2,3]. They also participate in functions such as phagocytosis and stress response [4]. This echoes Barry Halliwell’s [5] “All aspects of aerobic life involve free radicals and antioxidants—you cannot escape them nor should you wish to”.

Free radicals are molecules or atoms characterized by possessing an unpaired electron that is always seeking a counterpart to reside inside the parent home or in the home of the other electron. Free radicals can be produced during cellular processes or as a result of the interaction of free radicals, reactive species, or free radical-generating systems with other molecules [6,7]. Many of the ROS and RNS are free radicals. There are also macromolecular radicals that are formed by the effect of oxygen, drug-induced free radicals, irradiation or free radicals on such macromolecules as protein and lipids. For example, it has been demonstrated that γ-irradiated proteins produce electron paramagnetic resonance (EPR)—detectable superoxide and huydroxyl radicals, in the presence of ferrous iron, via their intermediate conversion to hydroperoxydes [6]. Such macromolecular radicals lead to cellular dysfunction and decreased cell viability [8]. In the presence of hydrogen peroxide, aminoglutethimide (xenobiotic) modified myeloperoxidase, transforming this enzyme in a macromolecular free radical; a reaction that has been suggested to have a role in the development of drug-induced agranulocytosis and has been inhibited by polyunsaturated fatty acids [7]. Generation of lipid-derived free radicals was demonstrated, by *in vivo* EPR, in the livers of rats treated with the carcinogen diethylnitrosamine [9]. Lipid-derived free radicals have also been demonstrated, through EPR, in culture cells incubated with ferrous iron [10]. Free-radical modified lipids [11] and lipid-derived free radicals may play an important role in the development of atherosclerosis.

**Figure 1:**
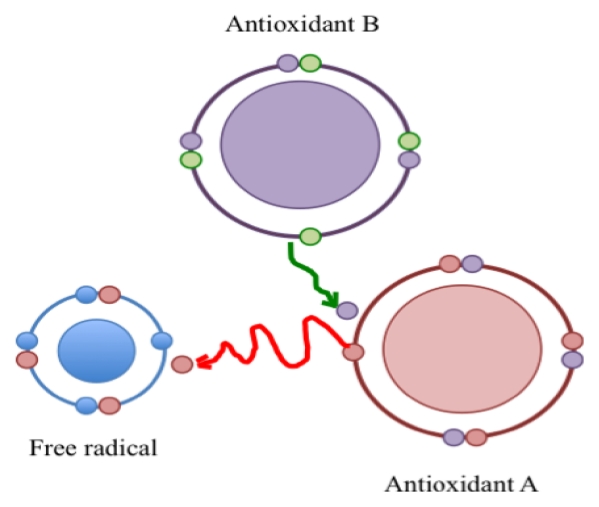
A free radical is a reactive species because it has an unpaired electron. Its reactivity is mitigated by an antioxidant (**A**) that donates an electron and in turn is converted to a reactive species that is recycled by a second antioxidant (**B**).

According to Halliwell and Gutteridge [12] an antioxidant is “any substance that, when present at low concentrations compared to those of an oxidizable substrate, significantly delays or prevents oxidation of that substance”. Just as reactive species are not necessarily bad, antioxidants are not necessarily good. Antioxidants are a kind of matchmaker midwife trying to give or receive an electron to complete the other’s “pair”, but by making a proper pair, antioxidants can themselves become incomplete, and behave as free radicals; *i.e.*, reactive and looking to pair with an unpaired electron. It is known that those “reactive” antioxidants can be stabilized (recycled) by other antioxidants, which in turn become “reactive” and can be recycled in a cascade of “reactivity mitigation” (Figure 1). It is all a matter of equilibrium. It well is accepted that oxidative stress is produced when the equilibrium between reactive species and antioxidants is tilted in favor of the former. However, the equilibrium can be broken also if antioxidant levels exceed those of the reactive species. Dündar [13] proposed the term “antioxidative stress” for such disequilibrium.

**Figure 2:**
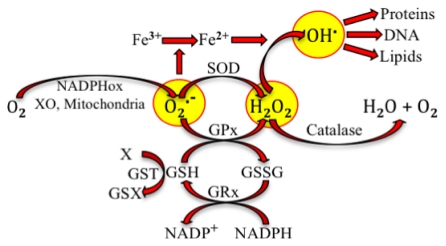
Superoxide radical (O_2._^−^) is produced from oxygen (O_2_) at complex I and complex III of mitochondria during respiration. It can also be produced through reactions catalyzed by NADPH oxidase (NADPHox) or xanthine oxidase (XO). Superoxide dismutase (SOD) and glutathione peroxide (GPx) catalyze the conversion by superoxide into hydrogen peroxide (H_2_O_2_, a reactive oxygen species). GPx requires the oxidation of glutathione (GSH) to oxidized glutathione (GSSG), which is reduced to recycle GSH through the enzyme glutathione reductase (GRx). GSH could be also used in detoxification (metabolism of the toxic X by conjugation with GSH) catalyzed by glutathione S transferase (GST). Superoxide participates in the reduction of ferric iron. Hydroxyl radical (OH.) is formed by the interaction of H_2_O_2_ and ferrous ion. OH. oxidizes proteins, DNA and lipids.

Nature is certainly wise. Aerobic organisms mitigate reactive species not only with antioxidant molecules that donate or receive electrons but also through reactions catalyzed by antioxidant enzymes. Figure 2, below, illustrates the production of superoxide by pro-oxidant enzymes and the intervention of antioxidant enzymes to stabilize such free radical.

Figure 2 is only an example of the different reactions that produce reactive species, which potentially lead to oxidative stress. We have taken this very simple sequence of reactions to illustrate how changes can lead to oxidative stress and make a later parallel with the antioxidant-induced stress. Superoxide radical production can be increased if the activity of NADPHox or XO increases. Indeed, it has been postulated and partially shown, that oxidative stress in hypertension is due, at least in part, to the increase of NADPHox’s activity in vascular smooth muscle cells, fibroblasts and endothelial cells [14,15]. Polymorphisms of the genes codifying SOD and GPx have been related to oxidative stress due to decreased activity of the enzymes and high incidence of coronary artery disease [16], ischemic stroke [17,18] and atherosclerosis [19]. Polymorphisms of GST have been associated with cardiovascular events produced in smokers [20].

## 2. Physiological Effects of Reactive Species

Reactive species participate in different functions and play a role as signal transduction elements in many physiological events. ROS and RNS participate in signal transduction of cytokine receptors, tyrosine receptor, serine/threonine kinases, G protein-coupled receptors, ion-channel linked receptors in response to angiotensin II, cytokines, glutamate, epidermal growth factor, vascular endothelial growth factor, tumor necrosis factor α and platelet derived growth factor [2]. Free radicals and ROS participate in the redox regulation of activator protein 1, nuclear factor κ B (NFκ-B), cyclic response element-binding protein (CREB), nuclear factor E2-related factor 2 (Nrf2) and p53 (“guardian of the genome”) [2,15]. Superoxide and hydrogen peroxide activate extracellular signal-related protein kinase (ERK), protein kinase B, mitogen-activated protein kinases (MAPKs) and insulin receptor kinase [15,21–23].

**Figure 3:**
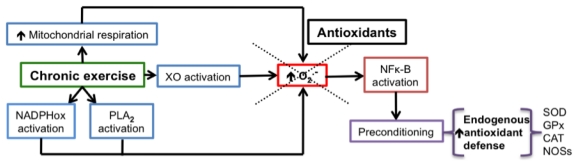
Chronic exercise increases the production of superoxide radical (O_2._^−^) through the increase of mitochondrial respiration and activation of NADPH oxidase (NADPHox), phospholipase A_2_ (PLA_2_) and xanthine oxidase (XO). The superoxide radical activates Nuclear Factor κ-B (NFκ-B), which in turn produces preconditioning (increase of the endogenous antioxidant defense), characterized by the increased gene expression and activity of superoxide dismutase (SOD), glutathione peroxidase (GPx), catalase (CAT) and nitric oxide synthases (NOSs). Antioxidants interfere with the effects of chronic exercise. More details are in references [34–39,41–43].

The effects of reactive species are dose-dependent. Incubation of Jurkat T-cells with increasing concentrations of H_2_O_2_ produced proliferation (0.7 μM), apoptosis (1.0–1.3 μM) or necrosis (>3 μM) [24]. ROS participate in proteolysis into the proteasome. H_2_O_2_ increases proteolysis at concentrations of 20–400 μM, whereas it inhibits proteolysis at higher, mM concentrations, producing an accumulation of oxidized proteins [25]. Preconditioning is one of the areas where the dose-dependent physiological effects of reactive species are evident. Repeated or unique application of injurious stimuli, at intensities below the threshold of damage, activates endogenous mechanisms that afford protection when subsequent major injurious stimuli are applied. This phenomenon is known as preconditioning [26]. Hyperbaric oxygen [26], short ischemia [27–29], small doses of lipopolysaccharides [30], restraint stress [31], hypoxia [32], hyperthermia [32] and moderate aerobic exercise [33,34] are some of the stimuli producing preconditioning. We chose the example of exercise to explain the role of reactive species on preconditioning (see Figure 3). An excess of ROS are produced during acute or intense exercise [35,36]. It was thought that mitochondria were the primary site of superoxide production during exercise, because oxygen consumption in respiration is increased. However, it is now known that superoxide is produced mainly through NADPHox, XO and phospholipase A_2_ (PLA_2_) during intense exercise [34]. Chronic moderate exercise suppresses oxidative stress produced by acute intense exercise [34,35]. The mechanism of such adaptation seems to involve, as has been demonstrated in different studies, activation of NFκ-B by ROS and the consequent expression of nitric oxide synthases and antioxidant enzymes [37–39]. Indeed, the incubation of cells with hydrogen peroxide induces the expression of antioxidant enzymes [40].

## 3. Pro-Oxidant Effects of Antioxidants

As mentioned above, antioxidants become “unstable” and “reactive” when they lose or receive electrons in the presence of reactive species (see Figure 1). In these conditions antioxidants exhibit pro-oxidant effects and can become harmful. Their redox potential (tendency to acquire electrons and thereby be reduced) could be related to the toxic effects of antioxidants. The redox potentials of some antioxidants of interest are: 0.65 V for β-carotene, 0.50 V for Vitamin E, 0.25–0.50 V for flavonoids, 0.25 V for uric acid and 0.01 V for Vitamin C [44–46]. It has been said that a network of antioxidants is necessary. Antioxidants should be interconnected in such a way that they can turn off each other’s reactivity after interacting with reactive species [47]. Precisely; Damiani [48] defined a good antioxidant as one that produces a low oxidant reactivity with a low capacity to produce peroxidation.

α-tocopherol (α-TC, Vitamin E) produces α-tocopheroxyl radical (α-TC.) when it reacts with reactive species such as peroxynitrite [49] or superoxide [50]. α-TC. is then recycled to α-TC by other antioxidants such as ascorbic acid (Vitamin C) and glutathione [48,51,52]. As soon as ascorbic acid recycles Vitamin E, it is transformed to the ascorbyl radical, which has a lower reactivity than α-TC. [48]. α-TC. is also recycled to α-TC by β-carotene [52,53]. Therefore, it is important that Vitamin E be administered with another antioxidant. It is also important to have significant levels of endogenous antioxidants to allow for the recycling of “reactive” antioxidants. Some conditions reduce endogenous antioxidants and could potentially affect the recycling of Vitamin E (or another antioxidant), leaving α-TC. available to produce lipoperoxidation. One such condition is the reduction of ascorbic acid in smokers [48,52]. Ascorbic acid also recycles oxidized glutathione (GSSG) to reduced glutathione (GSH) [51], and carotenoid radicals to carotenoids [53]. Dihydro-lipoic acid (DHLA), the reduced form of lipoic acid (an antioxidant) recycles GSH from GSSG and the ascorbic ascorbyl radical [54].

The pro-oxidant or antioxidant effects of some antioxidants depend on their concentration. For example lipoic acid and DHLA inhibit nitro-tyrosine formation by peroxynitrite at a 0.01–0.05 mM concentration, whereas promote mitochondrial permeability transition at 0.1 mM, increase of glucose intake at 0.25 M, and stimulate calcium mitochondria release at 1 mM. β-carotene protects DNA oxidative damage at 1–3 μM, whereas it oxidizes DNA at 4–10 μM. High concentrations of β-carotene inhibit the growth of cancer cells in culture and promote their apoptosis [53].

Some antioxidants exhibit pro-oxidant activities in the presence of transition metals. Such is the case of ascorbic acid that is transformed into an ascorbyl radical [55] and hydroxycinnamic acid whose phenolic groups are transformed into phenoxy reactive groups [56]. Retinal and carotenoid radical are produced by the oxidative cleavage of β-carotene in the presence of oxygen and ferrous ion [57,58]. Oxidative cleavage of β-carotene is also produced by many other oxidative stimuli: ultraviolet light (artificial and sun light), heat, free radicals, and ozone [59]. Carotenoid breakdown products (CBP) were produced in ferrets by cigarette smoke exposure. The effect was prevented by α-TC and ascorbic acid [52].

Reactive species produced by the interaction of an antioxidant with a free radical such as ROS or RNS exhibit potentially toxic pro-oxidant effects. CBP inhibit mitochondrial respiration in the rat lung, brain and liver [59], stimulate apoptosis in neutrophils [59], oxidize DNA [60] and up-regulate advanced glycation endproducts [53].

In summary, the pro-oxidant and potential harmful effects of an antioxidant depend on (a) its concentration; (b) its redox potential; (c) the presence of other antioxidants; (d) the presence of transition metals, (e) the activity and concentrations of endogenous antioxidants. And, even though not yet demonstrated, the genetic background could also contribute to the harmful effects of antioxidants. Some of these factors are further considered in the following section.

## 4. Clinical Trials with Antioxidants

The validity of antioxidant therapy has been questioned, having been pointed out that even though several diseases have been related to oxidative stress, antioxidant therapy does not change the natural course of diseases [5,47,61–64]. Some authors have commented that antioxidant therapy has only been validated in experimental models of disease and cell culture [5,61]. However, different factors cause artifacts in cell culture (e.g., high oxygen levels compared to the pO**_2_** of tissues in the organism, components of the cell culture media, high concentrations of antioxidants) [5,61]. In the clinical arena one of the problems is the biomarkers to evaluate oxidative stress [63]. It has been postulated that a combination of low doses of antioxidants could only be useful in patients suffering from antioxidant deficiencies [63]. It remains unknown if patients with gene polymorphisms affecting the structure and function of antioxidant enzymes could have different responses to antioxidants.

What is interesting is that when no effects or negative results of antioxidants are reported, there is often an alternate conclusion that antioxidants can be dangerous. For example: the method used to evaluate oxidative stress, treatment strategy (preventive strategy, doses, combination of antioxidants, duration), the stage of the disease, the follow up period, and number of patients [65]. Interestingly, in a recent review, Ristow and Schmeisser [66] suggest that life span could be extended by oxidative stress. A summary of clinical trials with antioxidants, as well as possible explanations are shown below.

### 4.1. Beneficial Effects of Antioxidants

Nurses’ Health Study (NHS). This was a cohort study of more than 87,000 U.S. nurses, ages 34–59, with no history of cardiovascular disease (CVD). Follow up period: 8 years. Results: Supplemental Vitamin E (at least 100 IU/day, for at least 2 years) was associated with reductions of 40% or more in the risk of coronary heart disease (CHD) [67,68].

Health Professional Follow up Study (HPFS). This was an observational study of around 40,000 U.S. male health professionals, ages 50–75, with no CHD, diabetes or hypercholesterolemia. Supplemental Vitamin E (at least 100 IU/day, for at least 2 years) was associated with a significant reduction of CHD risk [67,69].

The “Established Populations for Epidemiologic Studies of the Elderly” program. This involved 11,178 U.S. men and women, ages 67–105. Results: There is a significant decreased risk of CHD mortality among those taking vitamin supplements [67,70].

The first National Health and Nutrition Examination Survey (NHANES I). This involved 11,348 U.S. women and men, ages 25–74. Conclusion: Vitamin C intake was inversely associated with all causes of mortality and CVD in men, but not in women [71].

Scottish Heart Health study. This involved 3833 women and 4036 men, ages 40–59, with no heart disease at the beginning of the study. Vitamin C and β-carotene intake were associated with reduced CHD, only in men [71,72].

In a recent meta-analysis Myung *et al*. [73] evaluated 22 case-control reports (from 274 articles), including 10,073 patients with a cervical neoplasm risk. They found preventive effects of Vitamins B**_12_**, C, E and β-carotene on cervical neoplasms.

Alpha tocopherol, Beta carotene cancer prevention study (ATBC). This involved 27,111 Finnish male smokers, ages 50–69. Follow up: 16–19.4 years. Results: A higher αTC intake was associated with a lower pancreatic [74] and prostate [75] cancer risk. In the same study high flavonoid intake was also related to lower pancreatic cancer risk [76].

### 4.2. Antioxidants Do not Change the Evolution of Diseases Related to Oxidative Stress

20,536 patients with hypertension at high cardiovascular risk were followed for five years. Treatment with ascorbic acid, Vitamin E and β-carotene neither reduced blood pressure nor changed mortality or morbidity [14,77].

Rotterdam study. This involved 4,802 Dutch men and women, ages 55–95, with no history of myocardial infarction (MI). Follow up: After 4 years, no association was observed between Vitamin E intake and the risk of MI [71,78].

Scottish Heart Health Study (see above). No protection was associated with Vitamin E. Vitamins C, E and β-carotene had no effect on all-cause mortality [71].

Heart Protection Study. This included 20,536 men and women with CHD or diabetes, ages 40–80. Follow up: After 5 years, the intake of Vitamin E (600 mg/day), Vitamin C (250 mg/day) and β-carotene (20 mg/day) had no effect [71].

Primary Prevention Project. This program involved 4,495 men and women, with an average age of 64 and at least one risk factor for CHD, who took a low dose of aspirin (100 mg/day) and Vitamin E (300 mg/day). The study was stopped early because other trials had demonstrated protection with aspirin. Vitamin E had no effect [71].

Heart Outcomes Prevention Evaluation Study (HOPES). This included 2545 women and 6999 men, ≥55, with CHD or diabetes and one risk factor for atherosclerosis. They received Vitamin E (400 IU/day) or a placebo, angiotensin-converting enzyme inhibitor or a placebo. A follow up after 4.5 years indicated that Vitamin E had no effect [71,79].

Gruppo Italiano per lo Studio della Sopravvivenza nell’Infarto Miocardico (GISSI). This included 11,324 Italian men and women who had survived a myocardial infarction within the 3 previous months. They received Omega-3 oils (1 g/day) and/or Vitamin E or no treatment. A follow up after 3–5 years showed that Vitamin E had no effect [71,80].

Rytter *et al.* [81] studied 40 patients with type 2 diabetes, who were randomly assigned to one of two groups, one treated with antioxidants extracted from fruits, berries and vegetables (tocopherols, carotenoids and ascorbate), and the other treated with placebo. The authors found that 12 weeks of treatment did not change the metabolic profile, neither oxidative nor inflammatory biomarkers, even though antioxidant systemic levels had increased. Recently, Suksomboon *et al.* [82] analyzed 9 trials including 418 type 2 diabetic patients who were treated with Vitamin E for 8 weeks. They found that Vitamin E did not improve glycemic control unless the patients began their treatment under bad metabolic control and with a Vitamin E deficiency.

Arain *et al.* [83] analyzed four clinical trials to evaluate the effects of Vitamin E on colorectal cancer. The trials included 94,069 patients (47,029 received Vitamin E) and had a follow up period of 4 years. The authors concluded that Vitamin E had no effect on colorectal cancer.

### 4.3. Harmful Effects of Antioxidants

We summarize here some of the published studies that show the harmful effects of antioxidant supplements in humans.

In a study made in Australia, 69 hypertensive patients with an ambulatory systolic pressure of >125 mmHg received treatment with Vitamin C (500 mg/day) and grape seed polyphenols (1000 mg/day) for 6 weeks. At the end of the treatment their systolic and diastolic pressures increased and there were no changes in their endothelium dependent vasodilation or oxidative stress biomarkers [84]. HDL Atherosclerosis Treatment Study (HATS). 160 men (<70) and women (<63), with low HDL and triglycerides <400 mg/dL. Patients were assigned to one of 4 groups: Simvastatin + niacin with or without Vitamin E (800 IU/day), Vitamin C (1000 mg/day), β carotene (25 mg/day) and selenium (100 μg/day); Antioxidants or placebo. A follow up after 3 years showed that antioxidant treatment blunted HDL elevation produced by simvastatin + niacin [71,85].

The Prostate, Lung, Colorectal and Ovarian cancer Screening Trial (PLCO) was a prospective study of 25,400 postmenopausal U.S. women, ages 55–74, who were followed for 10 years. The results showed that the risk of breast cancer increased significantly (by 20%) in women who had a folic acid supplement (≥400 μg/day) whereas food folate intake was not associated with an increased risk [86]. Beta carotene and Retinal Efficacy Trial (CARET). This involved 18,314 U.S. women and men on β carotene (30 mg/day) and Vitamin A (25,000 IU/day) or a placebo. The study had to be stopped two years prematurely because Vitamin-treatment was associated with a 28% greater incidence of lung cancer and 17% more deaths than placebo treatment [71,87]. Alpha Tocopherol Beta Carotene Cancer Prevention Study (ATBC). This covered 29,133 male smokers, ages 50–69, taking α-TC (50 mg/day) and/or β-carotene (30 mg/day). There was an 8% greater mortality in those men taking the supplements than the men treated with the placebo [71,88,89]. Bjelakovic *et al.* [90] published a meta-analysis of antioxidant supplements for the prevention of gastrointestinal cancer. They selected 7 high quality randomized trials (of the 14 trials examined), including 131,727 patients. They found that antioxidants significantly increased overall mortality and did not prevent gastrointestinal cancer. Myung *et al.* [91] analyzed 31 of 3,327 articles searched, including 22 trials with 161,045 patients (88,610 treated with antioxidants). The authors concluded that antioxidants had no preventive effects on cancer. However, when they evaluated a subgroup of four controlled trials they found that patients receiving antioxidants had a significant increase in bladder cancer.

Antioxidants Vitamin C (12.5 mg/Kg) and N-acetylcysteine (10 mg/Kg) significantly increased the oxidative stress produced by acute exercise in healthy subjects. The effect was attributed to the conversion of ascorbic acid into the ascorbyl radical by reactive species generated during exercise [41]. Ristow *et al*. studied insulin sensitivity, transcriptional regulators of insulin sensitivity and gene expression of SODs, GPx and catalase in 19 trained and 20 untrained healthy young men who were treated with Vitamin E (400 IU/day) + Vitamin C (1000 mg/day) or placebo, and were trained for four weeks. They found that exercise increased insulin sensitivity as well as the gene expression of insulin sensitivity transcriptional regulators and antioxidant enzymes. The effects of exercise were blocked by antioxidant supplements [42]. Recently, Peternelj and Combes [43] evaluated 23 studies that reported negative effects of antioxidant supplements on the beneficial effects produced by chronic exercise. They found that antioxidants interfere with vasodilation and increased insulin signaling produced by exercise.

Recently, the Iowa Women’s Health Study results were published [92]. The study included 38,772 women, who were older than 60 in 1986. Vitamin and mineral supplement intake was self-reported at different periods. The authors concluded that dietary Vitamin and mineral supplements may be associated with increased mortality, with the effect being worse when the supplements were accompanied by an iron supplement.

Table 1 summarizes the results of the clinical trials described above. Some data command attention. The total number of study patients reporting beneficial antioxidant effects is 194,734 whereas those reporting no, or harmful effects is 554,083 (149,097 and 404,986, respectively). After comparing the trials, it can be concluded that the results probably depended on the population studied (age, gender, health conditions, ethnicity), antioxidants (type, combinations, supplements or dietary), follow-up period, and specific outcome. For instance, comparing the NHS study (beneficial effects) [68], with the Iowa Woman’s Health Study (IWHS, harmful effects) [92], it is seen that, even though the number of participants (all women) is higher in the NHS study (more than double the IWHS), there are significant differences: participants in the NHS study were younger, the follow up period was shorter in the NHS study, the focus of the NHS study was vitamin E whereas the IWHS examined the effect of supplement and dietary vitamin/mineral, outcome in the NHS study was only cardiac heart disease, whereas outcome in the IWHS was mortality.

Similarly, comparing the HPFS study (beneficial effects) [69] with the ATBC study (harmful effects) [89], there are differences that may have influenced the results. The HPFS study was performed in healthy men, whereas the ATBC study was run with male smokers. The HPFS study examined the effect of Vitamin E; the ATBC study analyzed the effect of Vitamin E and β-carotene. The follow-up period was shorter in the HPFS study and the reported outcome was different: *i.e.*, risk of CHD in the HPFS study *vs.* lung cancer and mortality in the ATBC study. Therefore, the main differences between the HPFS and ATBC studies are the particular conditions of the participants and the antioxidants related to the results. It is known that smoking produces oxidative stress and cancer [93,94]; and also known, as mentioned above, that an antioxidant is converted to a reactive species when it interacts with a free radical (Figure 1). A second antioxidant, by reacting with the “activated” antioxidant, can mitigate its free radical effects and recycle it as an antioxidant. These could explain the results of the ATBC study. Vitamin E alone did not produce any change, but the incidence of lung cancer and mortality increased in those participants who received Vitamin E and β-carotene [89]. Moreover, the CARET study, that looked for effects of Vitamin A and β-carotene in women and men who had worked with asbestos or were smokers [87] (both conditions are related to cancer), had to be stopped prematurely because of the clear correlation of increased lung cancer incidence and mortality. It can be postulated that β-carotene, in the presence of severe oxidatively damaged of DNA, could contribute to genomic changes leading to cancer. Interestingly, β-carotene is one of the antioxidants with the highest redox potential [46]. This characteristic could be related to the potential toxic effect of β-carotene in the presence of severe OS. A high redox potential could make β-carotene more capable of oxidizing molecules. Indeed, high concentrations of β-carotene itself oxidize DNA [53]. It has been postulated that smoke carcinogens alter carotenoid metabolism, contributing to the effects of DNA mutation and proliferation [95].

Another mechanism that seems to affect the final result of antioxidant supplementation is the normal responses of the organism to OS. In the first part of this review, it was mentioned that acute exercise produces OS [34–39]. Chronic exercise reduces the OS produced during acute exercise by activating NFκ-B, which leads to gene expression of antioxidant enzymes, and NOSs [37–39]. The phenomenon is termed “preconditioning”. It has been postulated that antioxidant supplements, by blocking free radicals and therefore the signal-transduction pathways that they activate during chronic exercise, inhibit such preconditioning [42,43] (see Figure 3).

## 5. Conclusions

Even though antioxidants have been considered beneficial, a number of clinical trials have shown harmful effects. The most extreme results evidenced increased mortality in individuals taking antioxidant supplements. Although the mechanisms are not explained in those trials, the results suggest that disrupting the delicate balance between antioxidants and reactive species produces antioxidant-induced stress, if the antioxidants overwhelm the physiological production of reactive species.

Though these conclusions remain speculative, the best advice would be to ingest antioxidants from food sources rather than from self-prescribed supplements. The most probable way of increasing the endogenous antioxidant defense would be by practicing moderate aerobic exercise, as an every day healthy routine. Gene polymorphisms, which condition endogenous antioxidant deficiencies, would be the exception to such advice.

## Figures and Tables

**Table 1:** Clinical trials with antioxidants.

Studies reporting beneficial effects of antioxidants						

Study	*N*	Age (years)	Follow up period	Antioxidant	Main Outcome	References
Nurses’ Health Study (NHS)	87,245	34–59	8 years	Vitamin E	Significant reduction in CHD risk	[67,68]
Health Professional Follow up Study (HPFS)	39,910	40–75	4 years	Vitamin E	Significant reduction in CHD risk	[67,69]
Established Populations for Epidemiological Studies of the Elderly	11,178	67–105	6 years	Vitamin E with or without other Vitamins	Vitamin E associated with a significant reduction CHD risk	[67,70]
First National Health and Nutrition Examination Survey (NHANES I)	11,348	25–74	10 years	Vitamin C	Inverse correlation between Vitamin C and all-cause CVD death in men; not women	[71]
Scottish Heart Health Study	7869	40–59	10 years	Vitamins C, E and β-carotene	Significant reduction of CHD; only in men	[71,72]
Meta-analysis	10,073	18–90	NR	Vitamins C, E and B**_12_**, and β-carotene	Preventive effects on cervical neoplasms	[73]
Alpha-Tocopherol, Beta carotene Cancer Prevention Study (ATBC)	27,111	50–69	16–19.4 years	Alphatocopherol, β-carotene and flavonoids	Alpha-tocopherol was associated with reduced risk of pancreatic and prostate cancer. Flavonoids were associated with decreased risk of pancreatic cancer	[74–76]
Heart Protection Study	20,536	40–80	5 years	Vitamins C and E and β-carotene	No reductions in blood pressure, morbidity or mortality	[14,77]
Rotterdam Study	4802	55–95	4 years	Vitamins C and E and β carotene	No effects of Vitamin E on the risk of myocardial infarction	[71,78]
Scottish Heart Health Study	7869	40–59	10 years	Vitamins C and E and β-carotene	No effects on all-cause mortality	[71,72]
Primary Prevention Project	4495	64 (average)	3.6 years	Vitamin E and low-dose aspirin	Vitamin E had no beneficial effect. Trial terminated because other studies demonstrated the beneficial effect of aspirin on cardiovascular mortality	[71]
Heart Outcomes Prevention Evaluation Study (HOPE)	9544	>55	4.5 years	Vitamin E	No effect of Vitamin E	[71,79]
Gruppo Italiano per lo Studio della Supravvivenza nell’ Infarto Miocardico (GISSI)	11,324	59.3 (average)	3.5 years	Vitamin E and omega-3 oils	No effect of Vitamin E	[71,80]
Study on well controlled diabetic patients	40	61.9	12 weeks	Extracts of fruits and vegetables	No effect of the extracts	[81]
Meta-analysis including studies performed in Type 2 diabetic patients	418	20–80	8 weeks	Vitamin E	No effects on metabolic control	[82]
Meta-analysis	94,069	>49	7–10 years	Vitamin E	No effect on colorectal cancer	[83]
Randomized, double blind, placebo controlled study in hypertensive patients	69	62 (average)	6 weeks	Vitamin C and grape seed polyphenols	Increase of blood pressure and no effect on either endothelium dependent vasodilation or oxidative stress	[84]
Randomized study in patients with CAD	169	52 (average)	3 years	One statin, Vitamin C, vitamin E, β-carotene and selenium	Antioxidants blunted the effect of statins on HDL	[71,85]
Prostate, Lung, Colorectal and Ovarian Cancer Screening Trial (PLCO)	25,400	55–74	10 years	Folic acid	Folic acid supplementation significantly increased breast cancer	[86]
Beta Carotene and Retinal Efficacy Trial (CARET)	18,314	58 (average)	Stopped after 2 years	Vitamin A and β-carotene	Antioxidant treatment was associated with an increased incidence of lung cancer and mortality	[71,87]
Alpha- Tocopherol Beta-Carotene Cancer Prevention Study (ATBC)	29,133	50–69	8 years	α-tocopherol, β-carotene	Antioxidants increased the incidence and mortality of lung cancer	[88,89]
Meta-analysis	131,727	55 (average)	1–12 years	β-carotene, Vitamin A, Vitamin E, selenium	The antioxidant treatment did not prevent gastrointestinal cancer but significantly increased mortality	[90]
Meta-analysis	161,045	58.4 (average)	5.3–5.8 years	β-carotene, Vitamin C, Vitamin E, selenium	Increased risk of bladder cancer in 4 of 22 of the trials included in the analysis	[91]
Study in healthy men	14	24.4 (average)	7 days	Vitamin C and *N*-Acetylcysteine	Increase of oxidative stress produced by exercise	[41]
Study in healthy men	39	25–35	4 weeks	Vitamin C and Vitamin E	Antioxidants blocked the increase of insulin sensitivity and expression produced by exercise	[42]
Meta-analysis	338 *	NR	1 day– 6 weeks	Allopurinol, Coenzyme Q, Vitamins (C, E, B**_6_**, B**_12_**), α-lipoic acid, β-carotene, lutein, *N*acetylcysteine, Selenium and/or Zinc	Any of these effects (compared to groups treated with placebo): ↓ training induced improvement in physical performance, ↓ exercise-induced oxidative stress preconditioning, ↑ CK, ↑ inflammatory biomarkers, prevented beneficial effects on insulin sensitivity and expression	[43]
Iowa Woman’s Health Study	38,772	>60	14 years	Dietary vitamins and mineral supplements	May be associated with increased total mortality risk	[92]

CAD = Coronary artery disease, CHD = Coronary heart disease, CK = creatine kinase, CVD = Cardiovascular disease, HDL = high density lipoproteins, NR = not reported.

* Includes 34 from Childs’ [41] and Ristow’s [66] studies.
